# Patterns of case management and chemoprevention for malaria-in-pregnancy by public and private sector health providers in Enugu state, Nigeria

**DOI:** 10.1186/1756-0500-5-211

**Published:** 2012-07-06

**Authors:** Ogochukwu C Onwujekwe, Rebecca O Soremekun, Benjamin Uzochukwu, Elvis Shu, Obinna Onwujekwe

**Affiliations:** 1 Department of Pharmacy, University of Nigeria Teaching Hospital, Ituku-Ozalla, Enugu State, Nigeria; 2 Department of Pharmacy, Lagos University Teaching Hospital, Idi-Araba, Lagos State, Nigeria; 3 Health Policy Research Group, Department of Pharmacology and Therapeutics, College of Medicine, University of Nigeria Enugu-Campus, Enugu, Nigeria; 4 Department of Community Medicine, College of Medicine, University of Nigeria Enugu-Campus, Enugu, Nigeria; 5 Department of Health Administration and Management, College of Medicine, University of Nigeria Enugu-Campus, Enugu, Nigeria

**Keywords:** Malaria in pregnancy, Chemotherapy, Chemoprophylaxis, IPTp, Providers

## Abstract

**Background:**

Malaria in pregnancy (MIP) is a major disease burden in Nigeria and has adverse consequences on the health of the mother, the foetus and the newborn. Information is required on how to improve its prevention and treatment from both the providers’ and consumers’ perspectives.

**Methods:**

The study sites were two public and two private hospitals in Enugu, southeast Nigeria. Data was collected using a pre-tested structured questionnaire. The respondents were healthcare providers (doctors, pharmacists and nurses) providing ante-natal care (ANC) services. They consisted of 32 respondents from the public facilities and 20 from the private facilities. The questionnaire elicited information on their: knowledge about malaria, attitude, chemotherapy and chemoprophylaxis using pyrimethamine, chloroquine proguanil as well as IPTp with sulphadoxine-pyrimethamine (SP). The data was collected from May to June 2010.

**Results:**

Not many providers recognized maternal and neonatal deaths as potential consequences of MIP. The public sector providers provided more appropriate treatment for the pregnant women, but the private sector providers found IPTp more acceptable and provided it more rationally than public sector providers (p < 0.05). It was found that 50 % of private sector providers and 25 % of public sector providers prescribed chemoprophylaxis using pyrimethamine, chloroquine and proguanil to pregnant women.

**Conclusions:**

There is sub-optimal level of knowledge about current best practices for treatment and chemoprophylaxis for MIP especially in the private sector. Also, IPTp was hardly used in the public sector. Interventions are required to improve providers’ knowledge and practices with regards to management of MIP.

## Background

Pregnant women are very vulnerable and badly affected by malaria in Africa [1]. In Nigeria, malaria remains a major public health problem and is responsible for 30 % childhood mortality and 11 % of maternal mortality [2-4]. More than 60 % out-patient visits in Nigeria are due to malaria and it also accounts for 30 % of all hospital admission with nearly 110million cases and 300,000 deaths per year [3].

Malaria infection during pregnancy has adverse consequences on the health of the mother, the foetus and the newborn. These effects include spontaneous abortion, preterm delivery, low birth weight, still-birth, congenital infection and maternal death [5]. The risk of anaemia in both the mother and baby is higher in first and second pregnancies than in later pregnancies [1,6].

During the past decade more effective strategies for prevention and control of malaria in pregnancy (MIP) have been developed [7]. The control of MIP using drugs could either be in the context of treatment or prevention through chemoprophylaxis or intermittent preventive treatment of malaria in pregnancy (IPTp). The current recommendation to control MIP in areas of stable malaria transmission relies on prompt and effective case management of malaria, the use of intermittent preventive treatment of malaria in pregnancy (IPTp) with at least two doses of sulphadoxine-pyrimethamine (SP) and the use of insecticide treated nets (ITNS) [5]. In the context of chemotherapy, the national treatment guideline recommends the use of quinine in all trimesters and artemether-lumefantrine in second and third trimesters [2].

Initially, attempts were made to prevent malaria in pregnancy using antimalarials which involved regular administration of drugs throughout pregnancy in order to sustain protective blood levels (chemoprophylaxis) [8]. The drugs used were chloroquine, pyrimethamine and proguanil [9]. However, studies reported the emergence and spread of drug resistance and adverse outcomes from the drugs [10]. This ultimately led to the concept of IPTp.

Nigeria adopted IPTp as a strategy in 2005 to replace weekly prophylaxis [11]. IPTp involves the administration of full curative treatment dose of an effective antimalarial drug (now only SP) at pre-defined intervals during pregnancy. The drug is given irrespective of peripheral blood malaria parasite status. It is should be provided to all pregnant women after 16 weeks of pregnancy [9].

IPTp with SP is offered as a package through focused ante-natal care (ANC) and as a national protocol, SP is given free of charge to pregnant women through ANC services at public health facilities and non-governmental organisation (NGO) facilities [12]. The treatment doses are given at least one month apart in the second and third trimesters [13]. To ensure that a pregnant woman receives at least two doses, delivery of IPTp should best be linked to routine scheduled antenatal visits after quickening. The effectiveness of SP in improving birth weight, and reducing prevalence of preterm deliveries and maternal anaemia was reported in Nigeria [14,15].

However, the IPTp coverage in Nigeria is low [12]. Only 18 % pregnant women received an antimalarial drug for the prevention of malaria during the pregnancy, 11 % received at least one dose of SP and 7 % received two or more doses [12]. Hence, it is important to examine current levels of provision of IPTp and to understand the underlying reasons behind levels of provision in both public and private sectors.

The paper assesses the knowledge and attitude of providers about malaria-in-pregnancy and compares the extent of provision of chemotherapy and chemoprevention (including IPTp) in public and private hospitals. It also examines the level of acceptability and use of IPTp. The information will be useful for improved delivery of effective interventions in the treatment and prevention of malaria in pregnancy using drugs.

## Methods

The study area was Enugu metropolis (state capital), Enugu State, south-east Nigeria. The state is made up of seventeen local government areas (LGAs), five of which are largely urban. It has a population of about 3,367,837 people and lies in an area of approximately 8721.1sq km land. The temperature in the state ranges between 22.4^0^ C and 30.8^0^ C and the vegetation is tropical rain forest with two major seasons namely dry (November to April) and rainy (May to October). It has an annual rainfall of between 1520 mm to 2030 mm. Majority of the residents are subsistence farmers and petty traders.

The state’s health delivery is through a network of formal and informal private and public health facilities. Malaria transmission in the area is stable and holoendemic. However, there is a higher rate of transmission during the rainy season. The infant mortality rate is 110/1000 and total fertility rate 5.6 and population growth rate 2.83 % [16].

### Study sites

The study was conducted in two public and two private hospitals in Enugu metropolis. The hospitals were purposively selected because they were the two busiest public and private hospitals and so assured the collection of timely adequate data because of the high number of women that receive ante-natal services (ANC) there. The two public hospitals were the University of Nigeria Teaching Hospital (UNTH) Ituku-Ozalla and the Enugu State University Teaching Hospital (ESUTH). The private hospitals were the Mother of Christ Specialist Hospital and Mbanefo Specialist Hospital.

The University of Nigeria Teaching Hospital is a referral centre. The Obstetrics and Gynaecology (O&G) clinics in the hospital run concurrently with the ANC clinic from Monday to Friday. There are five O&G units and each comprises about six Consultants, 4 Senior Registrars Medical Officers, 2 Pharmacists and 7 Nurses providing antenatal care. The clinic attends to an average of about fifty antenatal women daily including about fifteen new registrations. The ANC clinics in Enugu State University Teaching Hospital hold from Mondays to Fridays except on Thursdays. The clinics are headed by two consultants assisted by senior registrars, house officers, a pharmacist and nursing staff. The clinics admit about twenty newly registered antenatal women daily and attend to about sixty old cases on antenatal days. The Mother of Christ Hospital is a faith based private/specialist hospital. It is an over 50 bedded hospital. It’s Obstetrics and Gynaecology unit has two consultants, 6 medical officers, nurses and a pharmacist in assistance. The antenatal clinic days holds on Mondays and Fridays with an average of seventy women including 20 new registrations seen daily. Finally, Mbanefo Specialist Hospital is a private hospital. It has two consultants that are assisted by medical officers, nurses and midwives. It attends to about 50 antenatal women and 10 new registrations on antenatal days.

### Data collection

The study population was made up of all healthcare providers that included doctors, pharmacists and nurses that were directly involved in provision of ANC services in the hospitals. The sample size was computed using a power of 80 % and confidence level of 95 % and assuming that a minimum of 15 % of health workers will be knowledgeable about IPTp. Epi Info software package was used for calculating sample size. All the consenting healthcare providers in the ANC services of each health facility were interviewed using a self-administered structured pre-tested questionnaire. The interviews were undertaken after obtaining the informed consent from the providers. The data was collected from May to July 2010.

The questionnaire was used to elicit information from the providers on: their facility, individual status, knowledge about malaria, attitude, practice of chemotherapy as well as chemoprophylaxis using pyrimethamine, chloroquine and proguanil and chemoprevention using IPTp.

Ethics approval was obtained from the University of Nigeria Teaching Hospital Ethics Committee. Each respondent also provided a verbal consent.

### Data analysis

The data from the public and private sectors were respectively merged to yield public and private sectors’ databases in Epi Info software package. Hence, data from UNTH and ESUTH were merged to provide data about public facilities and data from Mbanefo and Mother of Christ merged to provide data about private facilities. In data analysis, tabulations were used to examine the distribution of the various variables and cross-tabulations used to compare the variables between public and private facilities. The denominator in data analysis was the total number of providers in the public and private sectors respectively. SPSS and STATA software packages were used for data analysis. Chi-square statistics and Fischer’s exact tests were used where appropriate to investigate existence of statistically significant differences in variables between public and private sectors. The levels of statistical significance were examined using chi-square statistics and the threshold set at the 0.05 level.

## Results

### Description of the providers and their malaria knowledge and attitude in public and private facilities

A total of 32 and 20 questionnaires were administered to providers in the public and private facilities respectively. Most providers in the public (84.4 %) and private (100 %) hospitals considered malaria-in-pregnancy (MIP) to be a very serious illness (Table 1). Majority of the respondents in public (87.5 %) and private (70 %) hospitals recognized anaemia as an adverse effect of severe malaria. Low birth weight was recognized by 65.6 % of public providers and 25 % of private providers (p = 0,004) as adverse effects of MIP. It is worthy of note that only 59.4 % of public providers and 15 % of private providers stated that MIP could lead to death of a pregnant woman.

**Table 1:** General knowledge of malaria by public and private providers

General malaria knowledge	Public n (%) N = 32	Private n (%) N = 20	*X*2 (p-value)
Malaria in pregnancy is a very serious illness	27 (84.4)	20 (100)	3.37 (0.064)
**Adverse effects on the women**			
Tiredness	19 (59.4)	2 (10.0)	12.463 (0.001)
Death of baby	25 (78.1)	9 (45.0)	5.967 (0.02)
Anaemia	28 (87.5)	14 (70.0)	0.156 (0.81)
Death of woman	19 (59.4)	3 (15.0)	9.929 (0.002)
Low birth weight	21 (65.6)	5 (25.0)	8.125 (0.004)
**Malaria detection in patients**			
Physical examination	7 (21.9)	0 (0)	0.037 (0.87)
Symptoms recognition	25 (78.1)	9 (47.4)	5.075 (0.02)
Microscopic examination	21 (65.6)	13 (68.4)	0.042 (0.84)
Rapid diagnostic test	14 (43.8)	3 (15.8)	4.194 (0.04)
Self recognition	4 (12.5)	0 (0)	0.283 (0.67)

Table 1 also shows how the providers stated that MIP could be diagnosed. In the public sector, a majority of the providers (78.1 %) knew that malaria could be diagnosed using symptoms as against 47.4 % in the private sector. However, 65.6 % and 68.4 % of public and private providers stated that microscopic examination could be used to diagnose malaria. Also, whilst 43.8 % of public providers knew that RDTs could be used to diagnose malaria, the proportion in the private sector was 15.8 %.

### Attitude of providers regarding malaria-in-pregnancy (MIP)

Most public and private providers strongly agreed that MIP was high risk factor and responsible for maternal and neonatal death, low birth weight and anaemia. However, whilst 15 (75 %) of private providers strongly agreed that the incidence of MIP was still on the increase despite interventions and polices, it was only 9 (28.1 %) of the public providers that strongly agreed with the assertion (p = 0.009). A total of 9 (45 %) of the private providers believed that daily or weekly chemoprophylaxis was still in use and very beneficial as against 3 (9.4 %) of the public providers (p = 0.014).

### Nature, practice and provision of chemotherapy by providers

It was found that 27 (84.4 %) of the public and 18 (90 %) private providers used blood tests to confirm the diagnosis of MIP. This was followed by the use of RDT, which was used by 7 (21.9 %) and 2 (10 %) of public and private providers respectively. In the public sector, the most common drug in general treatment of MIP was ACT 22 (68.8 %), followed by quinine 16 (50.0 %). Conversely, in the private sector, the most common drug was SP 14 (70.0 %) followed by ACT and quinine 5 (25.0 % respectively). Also, in treating MIP in the first trimester, 11 (34.5 %) of the public providers gave quinine while 3 (15.0 %) gave the drug in the private sector (p = 0.01). Other drugs that were provided were chloroquine tablet by 7 (21.9 %) by the public providers and 3 (15 %) by the private providers (p = 0.72); and SP by 4 (12.5 %) of public providers and 13 (65.0 %) of private providers (p = 0.001).

### Perception and provision of chemoprophylaxis by providers

Table 2 shows that 35 % of the private providers and 18.8 % of public providers found that weekly chemoprophylaxis using pyrimethamine, chloroquine and paludrine were still very useful control measures for MIP. The table also shows that 50 % of the private providers and 25 % of public providers still used chemoprophylaxis. However, there was no statistically significant difference in the number of women that were provided with chemoprophylaxis in both sectors (p = 0.058).

**Table 2:** Level of providers’ perception and provision of chemoprophylaxis for malaria in pregnancy

Variables	Public n (%) N = 32	Private n (%) N = 20	Chi^2^ (p-value)
**Opinion of usefulness of Daraprim, Chloroquine, Paludrine for chemoprophylaxis**			
Very useful	6 (18.8)	7 (35.0)	
Useful	15 (46.9)	9 (45.0)	4.44 (0.22)
Not useful	5 (15.6)	0 (0)	
Don’t know	6 (18.8)	4 (20.0)	
No of providers that prescribed daraprim, chloroquine, paludrine to the women	8 (25.0)	10 (50)	8.92 (0.003)
No of women chemoprophylaxis was provided for	10(31.3)	13 (65.0)	3.597 (0.058)

### Knowledge and provision of intermittent-preventive treatment of malaria in pregnancy

It was found that most of the private and public sector providers respectively knew about IPTp (Table 3). However, whilst 73.7 % of private sector providers strongly agreed that IPTp should be given at least twice during the course of the pregnancy with the first after quickening, the proportion was 50 % in the public sector (p = 0.025). Table 3 also shows that 73.7 % of private sector providers strongly agreed that IPTp should be given irrespective of fever presentation as against 63.3 % by public sector providers. More private providers stated that IPTp was very useful compared to public providers.

**Table 3:** Knowledge of IPTp by providers

Variables	Public n (%) N = 32	Private (n (%) N = 20	Chi^2^ (p-value)
General knowledge about IPTp	29 (90.6)	20 (100.0)	1.89 (0.28)
**Meaning of IPTp**			
Preventive strategy with ACT	3 (9.4)	2 (10.0)	
Preventive Strategy with Quinine	0 (0)	1 (5.0)	2.55 (0.47)
Preventive strategy with SP	23 (71.9)	13 (65.0)	
Preventive strategy that replaced pyrimethamine, chloroquine and paludrine	3 (9.4)	4 (20.0)	
**Knowledge of timing**			
Strongly agree	15 (46.9)	14 (70.0)	9.32 (0.025)
Agree	13 (40.6)	1 (5.0)	
Disagree	1 (3.1)	3 (15.0)	
Don’t know	1 (3.1)	1 (5.0)	
**Benefit of direct observation in IPTp**			
Strongly agree	10 (31.3)	14 (70.0)	
Agree	10 (31.3)	3 (15.0)	9.15 (0.027)
Indifferent	6 (18.8)	0 (0)	
Don’t know	6 (18.8)	3 (15.0)	
**Benefits of Health Education to implement**			
Strongly agree	25 (78.1)	15 (75.0)	0.02 (0.89)
Agree	6 (18.8)	4 (20.0)	
**Usefulness of IPTp**			
Very useful	14 (43.8)	11 (55.0)	1.04 (0.60)
Useful	13 (40.6)	7 (35.0)	
Not useful	1 (3.1)	0 (0.0)	

Table 4 shows that 100 % of the private providers prescribed IPTp compared to 79.3 % of public sector providers (p = 0.34). The table also shows that among the providers in the public facilities 71 % recommended IPTp from 2nd trimester as against 66.7 % in private sector. Some providers in both sectors prescribed IPTp in the first trimester.

**Table 4:** Patterns of provision of IPTp by providers

Variables	Public n (%) N = 32	Private n (%) N = 20	Chi^2^ (p-value)
Provision of IPTp	23 (79.3)	20 (100)	4.49 (0.34)
**Best time for provision of IPTp**			
1st trimester	6 (19.4)	5 (27.8)	1.72 (0.79)
2nd trimester	22 (71.0)	12 (66.7)	
3 rd trimester	1 (3.2)	1 (5.6)	
Others	1 (3.2)	0 (0.0)	
Provision of direct observed IPTp	9 (32.1)	16 (80.0)	10.7 (0.001)
No of women provided with IPTp in one month	12 (37.5)	16 (80)	8.30 (0.004)

The main drug of choice for IPTp in both the public and private sectors was SP by 23 (71.9 %) by the public providers and 18 (90 %) of the private providers (p = 0.68). Artesunate monotherapy were used by 8 (25.0 %) of the public providers and none by private providers (p < 0.01). Other drugs that were prescribed for IPTp were chloroquine tablets by 3 (9.4 %) of the public providers and 2 (10.0 %) of the private providers (p = 0.74); quinine by 2 (6.3 %) of the public providers and 1 (5.0 %) of the private providers; and ACT by 7 (21.9 %) of the public providers and 2 (10.0 %) of the private providers (p = 0.27).

## Discussion

Knowledge of malaria among health providers was high in both public and private sectors, but the public sector providers conformed more to the WHO recommendation of using microscopy and rapid diagnostic tools for confirming malaria in pregnancy [3,17,18]. More of the public providers recognized the adverse effect of malaria-in-pregnancy (MIP), in line with the WHO malaria report. Some of these effects include severe anaemia, low birth weight, and intrauterine growth retardation which invariably could lead to death of the baby [2].

In the provision of chemotherapy, artemisinin-based combination therapy (ACT) and quinine were the most commonly used by the public providers, while SP was most commonly used in the private sector. In the first trimester, the drugs of choice for the public sector health providers were quinine, ACT and Chloroquine while SP, ACT and Quinine were the drug of choice in the private sector. In the second trimester, the drugs most commonly used in the public were ACT and SP while the private used SP and Quinine. In the third trimester, the drugs of choice were ACT and SP in both the private and public health facilities.

The fact that SP was commonly used for treatment rather than prevention by the private providers negates the concept IPTp, which involves the administration of a full therapeutic course of an antimalarial to pregnant women at specified times regardless of whether they are infected or not [9]. The finding that the drug of choice for treatment of malaria in the first trimester for the public providers was mainly quinine is in line with 2006 guidelines that regards this drug as a treatment option in the first trimester [19].

The public providers conformed to the 2010 WHO recommendation in the provision of chemotherapy in the second trimester. The use of ACT by majority of the public health providers is in line with the WHO recommendation. Because of the uncertainties of artemisinin containing drugs, they may not be used in the management of uncomplicated malaria in the first trimester. SP is not given early because of theoretical concerns over the association of folate antagonists with neural tube defects and other congenital abnormalities [20]. In the second trimester, the commonly used drugs in the public sector were ACT and SP in line with the WHO recommendation.

Hence, the practice and provision of chemotherapy by public sector providers conformed to the WHO guidelines for malaria treatment in endemic areas and this was consistent for the three trimesters. A study in Lagos state found that 53.7 % of prescriptions contained ACT, 28.4 % contained Chloroquine, 16.8 % contained SP and 0.4 % contained Quinine [21]. The current recommendation for treating severe malaria in high transmission areas is quinine or an artemisinin derivative [19,22]. However, it is recommended that more interventions are required to improve prescribing practices of providers, so that they will stop prescribing chloroquine and artesunate monotherapy for treatment of MIP.

There was irrational provision of services as the daily and weekly chemopropylaxis, with pyrimethamine, chloroquine and proguanil were still being used. The proportion of private providers giving weekly prophylaxis was still very high. It was of interest to find that many providers (although much more in the private sector) stated that chemoprophylaxis using pyrimethamine, chloroquine and proguanil were still very useful in the control of MIP and these drugs were still being prescribed. However, it is known that the usefulness of weekly chemoprophylaxis has been limited because of poor adherence by pregnant women to a weekly or more frequent drug regimen and also increasing levels of *plasmodium falciparum* resistance to the drugs [23]. A previous study that was undertaken in 2001 in Nigeria showed that 86.8 % out of 91 obstetricians routinely provided malaria chemoprophylaxis using chloroquine, pyrimethamine, proguanil, and sulphadoxine-pyrimethamine either singly or in various combinations to pregnant women. It could be speculated that the greater use of the chemoprophylaxis by private providers in this study could be due to lack of knowledge of their decreasing effectiveness. However, further research, especially using qualitative methods could be used to understand the factors behind provider behaviour in provision of chemoprohylaxis.

Concerning IPTp, it was reassuring that most of the providers in both sectors were quite knowledgeable about IPTp and recognized it as a preventive strategy. The fact that many providers were aware of the benefits of direct observation of the consumption of drugs is very important as many of the women when given the drug to take home may throw it away. Only 36.8 % of 57 women offered SP in a facility-based study took the drug [24]. A study in Korongwe district in Tanzania recommended that clean water, cups and soapy water be made available at facilities to further strengthen the direct observation strategy [25]. In a study in Gambia the direct observation of SP on an average of three occasions increased the compliance [9].

The private providers provided IPTp more to the pregnant women compared to the public providers. However, the timing of IPTp was better in the public sector compared to the private sector. It was found that some providers prescribed IPTp with SP in the first trimester, with the attendant risks to the foetus. SP was mostly used for IPTp although some public sector providers also used ACT. This is in contrast to a study carried out in UNTH, Enugu [8] where one tenth of Nigerian obstetricians surveyed did not routinely prescribe IPTp during pregnancy and majority of them relied on pyrimethamine, chloroquine and proguanil either singly or in combination.

The study has some limitations, which may have influenced some of the findings. The limitations include the purposive sampling and using only four healthcare facilities and the mix of providers in those facilities. It is possible that inclusion of lower level facilities such as primary healthcare centres and secondary level public hospitals would have provided a more holistic picture of nature of provision of treatment and prevention for MIP. Also, the non-disaggregation of data by types of providers (because of the limited sample size), constrained our understanding of influence of type of providers (doctors, nurses, midwives etc.) on case management and chemoprevention of MIP. Also, qualitative questions and open ended questions could have helped to elicit more information on the subject matter, instead of just close-ended questions that was used. It is also possible that because self-administered questionnaires were used, some respondents could have consulted each other on how and what to fill in the questionnaire.

Future studies should examine the underlying reasons between high ANC attendance and low IPTp provision, as well as why behaviour of public and private providers (including private providers more positive attitude to IPTp) different in some cases as was found in this study. Future studies should also incorporate qualitative research methods, so as to elicit more explanatory information concerning the nature of provision of treatment and prevention for MIP and disaggregate the data by different types of healthcare providers. Future studies could interviewer-administered questionnaires so that the biases that may arise from respondents consulting each other before completing the questionnaire will be limited.

## Conclusions

It is recommended that malaria control programmes should design and implement focused interventions that will improve prescribing practices of providers so that they will stop prescribing chloroquine and artesunate monotherapy for the treatment of MIP. Also increased Information Education and Communication (IEC) campaigns targeting consumers and providers as well as innovative training by National and State Malaria Control Programmes could be used to limit the provision of inappropriate drugs by providers for MIP. It is also important that both the public and private sector health providers should implement the direct observation of therapy (DOT) when using SP for IPTp. Patients should be provided with disposable cups at the facilities and clean water in order to strengthen the DOT strategy.

## Competing interests

The authors declare that they have no competing interests.

## Authors’ contributions

OCO, RS and OO conceived the study, participated in the design and performed the statistical analysis. BU and ES participated in the design, data collection and analysis. OCO drafted the manuscript. All the authors read and approved the final manuscript.
